# Supplementary material for: Identification of photoperiod-induced specific miRNAs in the adrenal glands of Sunite sheep (Ovis aries)
Source: Front Vet Sci. 2022 Jul 22;9:888207. doi: 10.3389/fvets.2022.888207 (PMC9354845; doi:10.3389/fvets.2022.888207)
Supplement: Supplementary file 1 [file Data_Sheet_1.ZIP › Supplementary file/miRDeep2 file.zip!/miRDeep2 file/Know_Structure/SP42A2/oar-mir-218a.pdf]

miRBase precursor : oar-mir-218a
Total read count : 53522
oar-miR-218a read count : 53520
remaining reads : 2

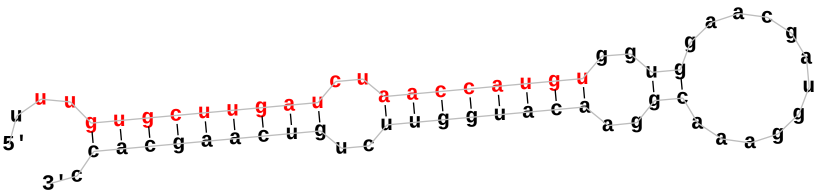

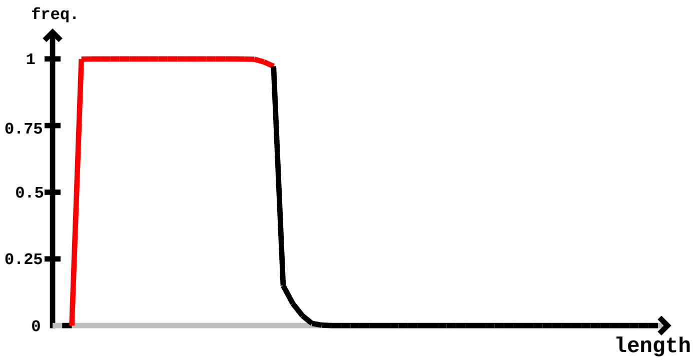


```
      oar-miR-218a
5'- uuugugcuugaucuaaccaugugguggaacgauggaaacggaacaugguucugucaagcacc   -3'    exp
    ...((((((((..((((((((..((............))..))))))))..))))))))).    reads      mm         sample
    uuugugcuugaucuaaccaugu........................................    3          0          seq
    .uugugcuugaucuaacA............................................    1          1          seq
    .uugugcuugaucuaacc............................................    28         0          seq
    .uugugcuugaucuaacca...........................................    39         0          seq
    .uugugcuugaucuaacAau..........................................    2          1          seq
    .uugugcuugaucuaaccau..........................................    536        0          seq
    .uugugcuugaucuaaccaug.........................................    865        0          seq
    .uugugcuugaucuaacAaug.........................................    6          1          seq
    .uugugcuugaucuaaccaugu........................................    43882      0          seq
    .uugugcuugaucuaacAaugu........................................    129        1          seq
    .uugugcuugaucuaaccaugug.......................................    3597       0          seq
    .uugugcuugaucuaaccaugugg......................................    2413       0          seq
    .uugugcuugaucuaaccauguggu.....................................    1555       0          seq
    .uugugcuugaucuaaccaugugguU....................................    313        1          seq
    .uugugcuugaucuaaccauguggug....................................    13         0          seq
    .uugugcuugaucuaaccaugugguUg...................................    90         1          seq
    .uugugcuugaucuaaccaugugguggg..................................    2          0          seq
    ..ugugcuugaucuaaccaugu........................................    6          0          seq
    ..ugugcuugaucuaaccaugug.......................................    1          0          seq
    ..ugugcuugaucuaaccaugugg......................................    25         0          seq
    ..ugugcuugaucuaaccauguggu.....................................    5          0          seq
    ..ugugcuugaucuaaccauguggug....................................    1          0          seq
    ...gugcuugaucuaaccaugu........................................    6          0          seq
    ....ugcuugaucuaaccauguggUUg...................................    1          1          seq
    .....gcuugaucuaaccauguggu.....................................    1          0          seq
    ......................gguggaacgauggaaacggaac..................    1          0          seq
    .......................guggaacgauggaaacggaaca.................    1          0          seq
```
